# An Alternative Model of Laser-Induced Stroke in the Motor Cortex of Rats

**DOI:** 10.1186/s12575-019-0097-x

**Published:** 2019-05-16

**Authors:** Matthew Boyko, Ruslan Kuts, Benjamin F. Gruenbaum, Philip Tsenter, Julia Grinshpun, Dmitry Frank, Vladislav Zvenigorodsky, Israel Melamed, Evgeni Brotfain, Alexander Zlotnik

**Affiliations:** 1 Division of Anesthesiology and Critical Care, Soroka University Medical Center and the Faculty of Health Sciences, Ben-Gurion University of the Negev, 84101 Beer-Sheva, Israel; 2 Department of Anesthesiology, Yale University School of Medicine, New Haven, CT 06525 USA; 3 Division of Internal Medicine, Soroka University Medical Center and the Faculty of Health Sciences, Ben-Gurion University of the Negev, 84101 Beer-Sheva, Israel; 4 Department of Radiology, Soroka University Medical Center and the Faculty of Health Sciences, Ben-Gurion University of the Negev, 84101 Beer-Sheva, Israel; 5 Department of Neurosurgery, Soroka University Medical Center and the Faculty of Health Sciences, Ben-Gurion University of the Negev, 84101 Beer-Sheva, Israel

**Keywords:** Stroke, Laser-technique, MCAO, model, Outcome, Variability, Cortex

## Abstract

**Background:**

A common experimental rodent model for stroke includes induction by a technique in which middle cerebral artery is transiently (MCAO-t) or permanently (MCAO-p) occluded by catheterization. However, this model has prominent disadvantages which consist of the high variability of localization and size of the ischemic area, cases of intracranial hemorrhage and high mortality. Furthermore, the duration of a single MCAO operation takes about thirty minutes and requires highly trained staff. In this article, we propose an alternative method, which is based on laser-induced stroke in the motor cortex. In our research, we compared the original MCAO-p and MCAO-t models and a novel laser model.

**Results:**

Compared with the impact of original MCAO-p and MCAO-t technique on brain tissue, the minimally invasive laser model demonstrated a decrease in: variability in body temperature, percent of infarcted volume, blood brain barrier breakdown and brain edema, as well as a prominent decrease of mortality and intracranial hemorrhage. Among other findings of this article, it can be noted that damage to the brain tissue in laser groups occurred only in the region of the motor cortex, without involving the striatal area.

**Conclusions:**

The data presented in this paper show that the model of laser irradiation can serve as an effective method of inducible brain cortical infarction and may lead to a better understanding of the pathophysiology of ischemic stroke and the future development of new drugs and other neuro-protective agents.

## Background

Stroke is the fifth most common cause of death worldwide after cardiac diseases, cancer, chronic lower respiratory diseases and accidents. Furthermore, stroke is the leading cause of serious long-term disability. Recent data demonstrate that one-fourth of stroke survivors eventually required transfer to skilled nursing facilities and approximately 30 % required inpatient rehabilitation or home health care [1].

Animal models of cerebral ischemia represent an important contribution both in the understanding of stroke pathophysiology and in the development of new therapies. Optimal model requirements consist of technical simplicity, low cost, high reproducibility and minimal variability in major outcomes of interest. In the case of inducible ischemic stroke models, the major outcomes of interest include infarct size, brain edema volume, blood brain barrier (BBB) breakdown extent and functional impairment estimated by neurological severity score (NSS).

MCAO procedure, which consists of transient or permanent middle cerebral artery occlusion, is the most frequently-employed method for experimental induction of ischemic stroke in rats and mice [2]. Currently, the intra-arterial suture occlusion technique is the most commonly accepted among the MCAO stroke models in rats. This model has prominent advantages: ischemic stroke origin similar to stroke in humans, presence of penumbra surrounding the induced stroke core, high reproducibility and precise control of duration of ischemia and reperfusion [3]. However, even this model has multiple limitations and complications such as high risk of intracranial hemorrhage, risk of ipsilateral retinal injury with visual dysfunction and common hyperthermia due to hypothalamic infarction leading to altered stroke outcome. Prominent disadvantages of this model include high variability in stroke size due to possibilities of ischemia in external carotid artery territory, inadequate middle cerebral artery occlusion and premature reperfusion. Furthermore, induced infarct volumes vary considerably between different strains and ages in mice and rats [4]. Marked variability in the degree of ischemia and volume of infarction has been a recurrent finding in this model, likely due to its relatively low resolution and inevitable variability in collateral vessels between different animals [5–7]. Another disadvantage of the MCAO procedure is that it cannot be used to induce small peripheral strokes because of the technical limitations of a minimum vessel size that can be catheterized. For the same reason MCAO cannot be used to induce small isolated strokes in deep brain areas. Furthermore, stroke inducement by MCAO model takes a relatively long time and requires a highly trained staff, because each middle cerebral artery catheterization is a relatively complicated operation which lasts about thirty minutes.

In our research, we propose a novel method of laser inducible stroke. Laser emits light through a process of optical amplification based on the stimulated emission of electromagnetic radiation. The laser device consists of a gain medium which can be solid, gaseous or liquid; an energy source; and a device which provides optical feedback. The mechanism of its action consists of turning the electrons of the gain medium to higher orbitals, causing the electrons of the gain medium to move to higher energy level. This excited state is induced by an external source of energy. Because the electrons always tend to return to lower possible energy level, they do so eventually and release energy consisting of photons. When the laser system continues to receive an external energy, most of the gain medium electrons stay excited, causing a massive release of photons from the material and causing other photons to release, causing an amplification effect. A system of mirrors serves as an optical resonator which makes possible numerous photon passages through the gain medium, enhancing the amplification effect and keeping most of the photon flow in a chosen direction. One of the mirrors permits partial passage of the photons which subsequently emits the laser beam.

This laser technique relies mainly on the photothermal effects of the laser on living tissue. This effect causes the absorption of the light beam into tissues of the body, especially blood, where it is converted into heat. The absorption by hemoglobin in the blood may coagulate or vaporize superficial vascular lesions, in addition to the direct damage to surrounding tissue by thermal destruction.

Current laser technology is multidisciplinary, easy to perform and safe. Laser has an ability to give up its energy, which is transmitted to a local heat at a chosen point, and this ability is used in medicine to stop bleeding during surgery. Another important characteristic of a laser is the ability of amplification of different beams when they meet at a chosen point. This method, which is used in radiosurgery, prevents healthy brain tissue damage from relatively weak laser beams which pass through and affect only a targeted point where the beams meet.

We wanted to use these laser abilities to construct an effective inducible stroke model in rats. The type of laser beam we used was able to pass through the medium with a low liquid content (bone) without giving up its energy and without causing destruction and did so only at medium which had a high liquid content (brain tissue). This way only brain tissue was affected. The laser beam could be sent in a chosen direction and thus induce a stroke at the precisely selected area of the brain. Another prominent laser advantage was the ability to regulate its level of irradiation and thus achieve our choice of different stroke severities. In addition, the ischemic area at the original MCAO model consists of cortex and striatum and the relative percentage of each of them cannot be predicted prior to the procedure. In a laser model we had the ability to regulate the power of the impact on the brain tissue and induce isolated motor cortex stroke without striatum involvement. For this reason, the laser model could be extremely useful in the study of isolated motor cortex ischemia.

In a more complicated model of laser inducible deep stroke (which was not investigated in this study) the desired stroke area is located at deep brain structures. In this case, it is possible to send multiple laser beams from different directions which are focused at a chosen point, similar to the technique used at radiosurgeries when brain tumors are removed without affecting the surrounding healthy tissue.

## Results

An important observation made at the study design stage determined that the minimal required laser power for inducement of a stroke at the level comparable to the level made by MCAO technique was 50X5 joules or more. Laser irradiation of lower power used in subgroups of 50X2, 50X1 and 25X1 joules did not induce a comparable level of strokes. But even in these subgroups the impact of the laser on the brain tissue was able to induce mild strokes and had a significant difference in comparison to the naïve subgroups.

**Fig. 1:**
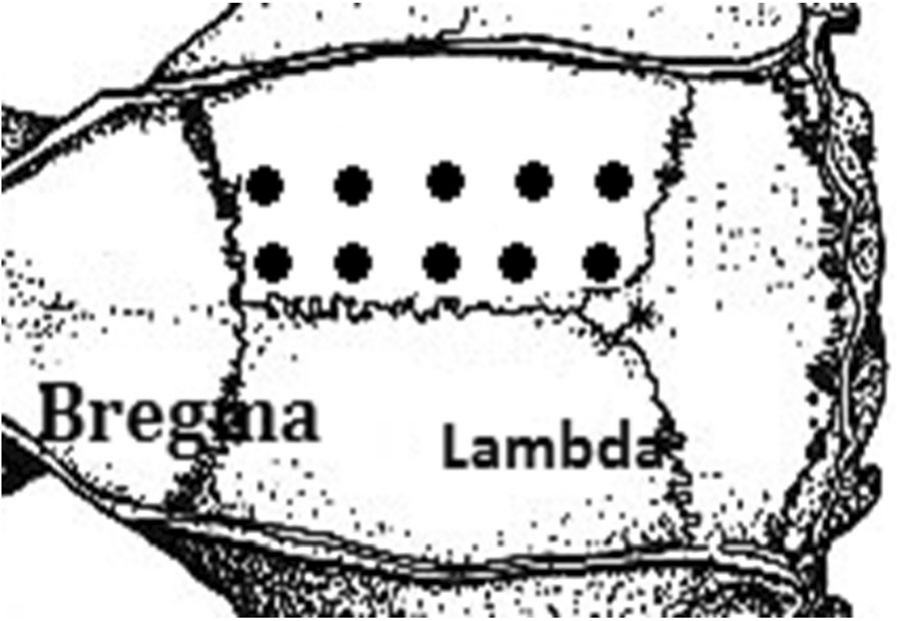
Targets for laser marked on the bone above the right hemisphere area

**Fig. 2:**
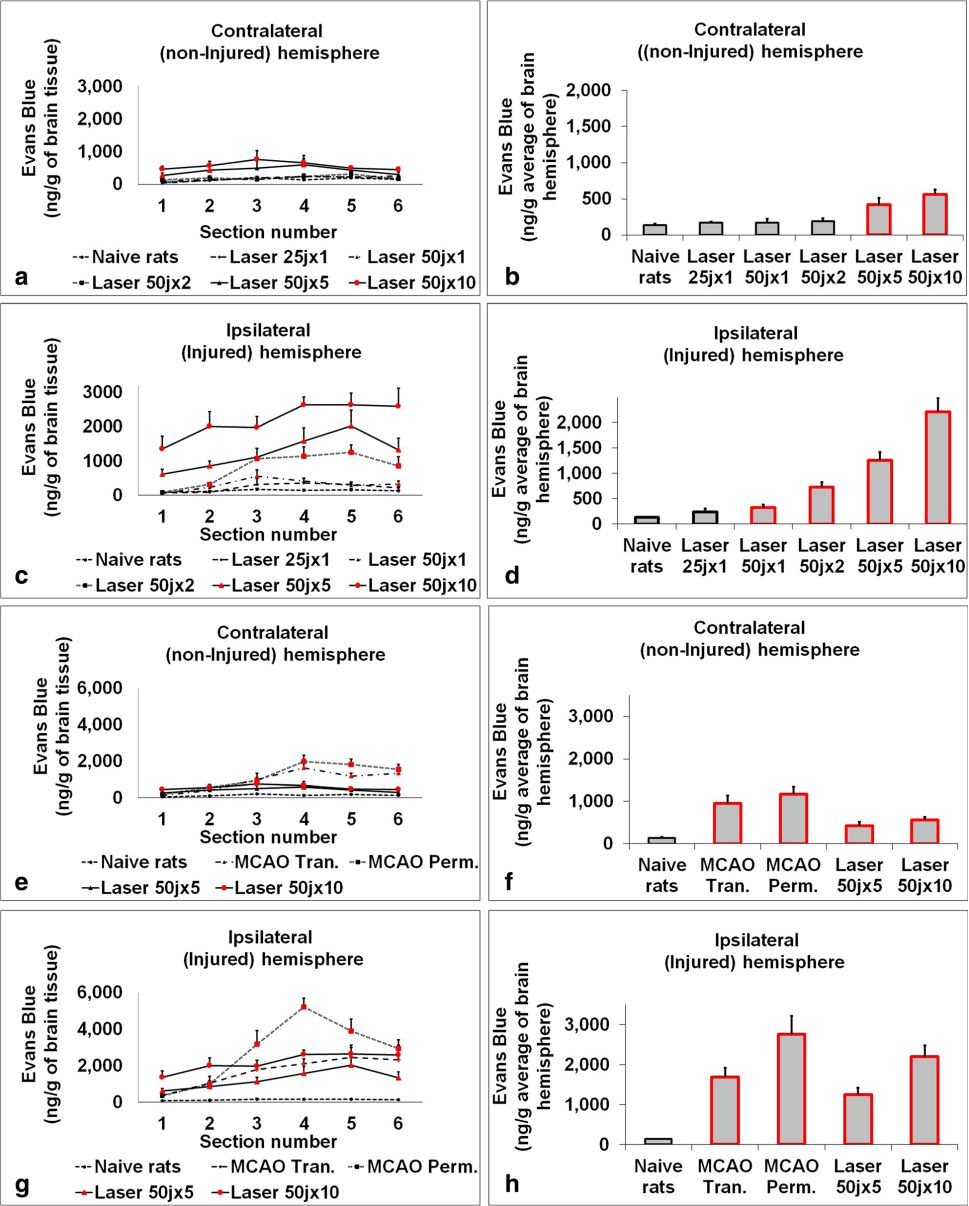
Illustrated BBB breakdown 24 h following MCAO or laser injury groups compared to naïve rats. In Fig. 2**a** shows the BBB breakdown in the non-injured hemisphere 24 h after laser procedure (presentation by sections). Comparison of laser groups with naive rats. In Fig. 2**b** shows the BBB breakdown in the non-injured hemisphere 24 h after laser procedure (presentation of the hemisphere average value). Comparison of laser groups with naive rats. In Fig. 2**c** shows the BBB breakdown in the injured hemisphere 24 h after laser procedure (presentation by sections). Comparison of laser groups with naive rats. In Fig. 2**d** shows the BBB breakdown in the injured hemisphere 24 h after laser procedure (presentation of the hemisphere average value). Comparison of laser groups with naive rats. In Fig. 2**e** shows the BBB breakdown in the non-injured hemisphere 24 h after laser and MCAO procedures (presentation by sections). Comparison of treatment groups with naive rats. In Fig. 2**f** shows the BBB breakdown in the non-injured hemisphere 24 h after laser and MCAO procedures (presentation of the hemisphere average value). Comparison of treatment groups with naive rats. In Fig. 2**g** shows the BBB breakdown in the injured hemisphere 24 hours after laser and MCAO procedures (presentation by sections). Comparison of treatment groups with naive rats. In Fig. 2**h** shows the BBB breakdown in the injured hemisphere 24 h after laser and MCAO procedures (presentation of the hemisphere average value). Comparison of treatment groups with naive rats. The data (Evans blue extravasation) are measured in ng/g of brain tissue and expressed as mean ± SEM. Red color indicates significance (*P* < 0.01)

**Fig. 3:**
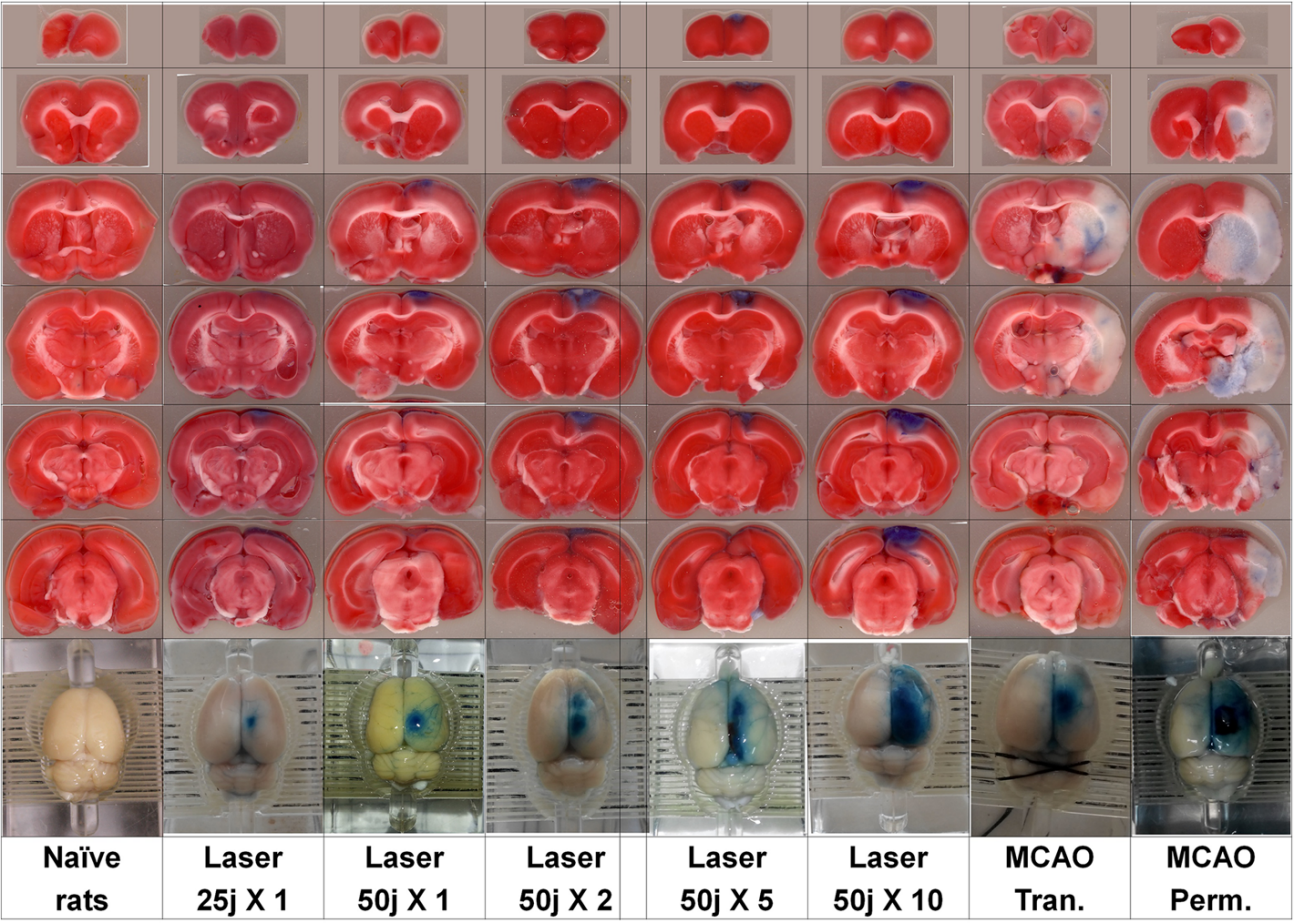
Histological cuts of rats’ brains from naïve, laser and MCAO subgroups (for illustration was used combined staining TTC and Evans blue)

**Fig. 4:**
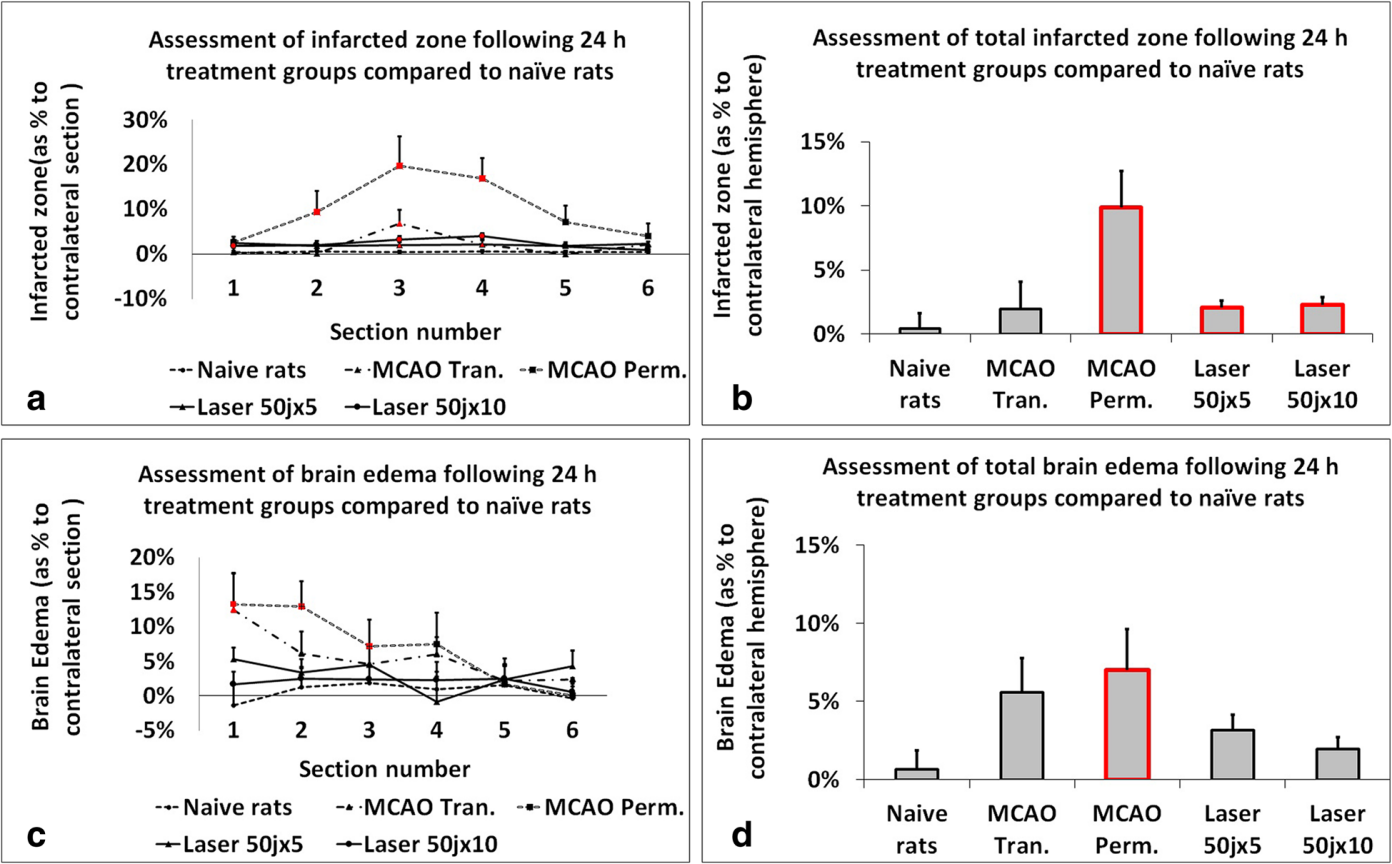
Illustrated assessment of infarct zone and brain edema 24 h following MCAO or laser injury groups compared to naïve rats. In Fig. 4**a** shows the assessment of infarcted zone following 24 h after laser and MCAO procedures (presentation by sections). Comparison of treatment groups with naive rats. In Fig. 4**b** shows the assessment of infarcted zone following 24 h after laser and MCAO procedures (presentation of the hemisphere average value). Comparison of treatment groups with naive rats. In Fig. 4**c** shows the assessment of brain edema following 24 h after laser and MCAO procedures (presentation by sections). Comparison of treatment groups with naive rats. In Fig. 4**d** shows the assessment of brain edema following 24 h after laser and MCAO procedures (presentation of the hemisphere average value). Comparison of treatment groups with naive rats. The data are measured as % to contralateral section/hemisphere and expressed as mean ± SEM. Red color indicate significance (*P* < 0.05)

**Fig. 5:**
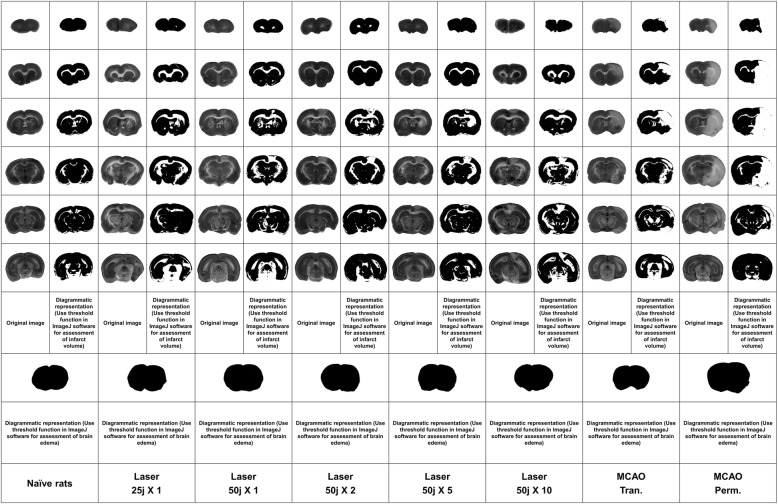
Distribution of ischemic damage and brain edema between the study groups

The data of the results of our research, as described in Figs. 1-5 and Tables 1 and 2 below, showed significant differences in some of the parameters of our major objects of interest between the laser and the MCAO subgroups.

**Table 1:** Assessment of infarct volume, NSS, body temperature, mortality and BBB breakdown in injured hemisphere. ^a^ = *p* < 0.01

Groups		BBB *n* = 10		Infarcted Volume *n* = 10		Temperature *n* = 20		Mortality n (%)
		Mean and SEM	Variability%	Mean and SEM %	Variability%	Mean and SEM °C	Variability%	
Naïve (*n* = 20)	1 section	80 ± 15	58	0.5 ± 0.3	191	37.2 °C ± 0.1	59	0%
	2 section	113 ± 20	55	0.6 ± 0.2	86			
	3 section	174 ± 33	60	0.4 ± 0.3	209			
	4 section	151 ± 22	46	0.6 ± 0.3	187			
	5 section	161 ± 33	65	0.4 ± 0.3	250			
	6 section	127 ± 15	37	0.6 ± 0.3	149			
	Total Hemisphere	134 ± 11	25	0.5 ± 0.1	77			
Laser 25jx1 (*n* = 20)	1 section	76 ± 36	148	1.1 ± 0.4	126	37.3 °C ± 0.1	61	0%
	2 section	83 ± 31	120	0.4 ± 0.3	215			
	3 section	315 ± 172	172	1.2 ± 0.4	97			
	4 section	362 ± 118	103	0.5 ± 0.4	252			
	5 section	310 ± 47	48	1.4 ± 0.4	74			
	6 section	212 ± 28	42	0.6 ± 0.3	163			
	Total Hemisphere	234 ± 67	90	0.9 ± 0.1	49			
Laser 50jx1 (*n* = 20)	1 section	53 ± 43	257	0.5 ± 0.2	142	37.3 °C ± 0.1	55	0%
	2 section	233 ± 90	122	0.9 ± 0.3	116			
	3 section	556 ± 180	102	0.7 ± 0.3	115			
	4 section	418 ± 88^a^	67	0.8 ± 0.2	84			
	5 section	281 ± 99	112	0.8 ± 0.3	97			
	6 section	326 ± 90^a^	88	0.5 ± 0.3	173			
	Total Hemisphere	321 ± 63^a^	63	0.7 ± 0.1	47			
Laser 50jx2 (*n* = 20)	1 section	89 ± 48	170	0.9 ± 0.2	81	37.3 °C ± 0.1	54	0%
	2 section	308 ± 67^a^	69	0.5 ± 0.3	203			
	3 section	1072 ± 295^a^	87	0.6 ± 0.4	199			
	4 section	1147 ± 260^a^	72	0.4 ± 0.3	223			
	5 section	1262 ± 207^a^	52	0.5 ± 0.3	170			
	6 section	853 ± 271^a^	100	1.1 ± 0.3	87			
	Total Hemisphere	725 ± 100^a^	44	0.7 ± 0.1	48			
Laser 50jx5 (*n* = 20)	1 section	617 ± 144^a^	74	2.3 ± 0.4^a^	51	37.4 °C ± 0.1	53	0%
	2 section	854 ± 145^a^	53	1.3 ± 0.3	71			
	3 section	1114 ± 249^a^	71	2.0 ± 0.4^a^	59			
	4 section	1585 ± 385^a^	77	2.8 ± 0.6^a^	63			
	5 section	2027 ± 460^a^	72	1.2 ± 0.3	90			
	6 section	1332 ± 340^a^	81	1.7 ± 0.5	89			
	Total Hemisphere	1255 ± 167^a^	42	1.9 ± 0.5^a^	33			
Laser 50jx10 (*n* = 20)	1 section	1359 ± 367^a^	85	2.2 ± 0.4^a^	58	37.4 °C ± 0.1	84	0%
	2 section	2011 ± 431^a^	68	2.4 ± 0.5	69			
	3 section	1977 ± 318^a^	51	3.3 ± 0.5^a^	46			
	4 section	2632 ± 234^a^	28	3.8 ± 0.6^a^	51			
	5 section	2644 ± 340^a^	41	1.6 ± 0.5	92			
	6 section	2600 ± 529^a^	64	1.3 ± 0.6	162			
	Total Hemisphere	2204 ± 280^a^	40	2.4 ± 0.3^a^	34			
t-MCAO (*n* = 25)	1 section	371 ± 211	179	0.5 ± 0.8	555	38.2 °C ± 0.1^a^	121	5 (17%)
	2 section	1053 ± 377^a^	113	0.1 ± 0.7	1618			
	3 section	1806 ± 507^a^	89	6.8 ± 3.0	141			
	4 section	2108 ± 262^a^	39	2.2 ± 1.3^a^	192			
	5 section	2649 ± 688^a^	89	−0.1 ± 0.8	4625			
	6 section	2338 ± 322^a^	44	2.2 ± 1	151			
	Total Hemisphere	1688 ± 231^a^	43	1.9 ± 0.8	123			
p-MCAO (*n* = 24)	1 section	355 ± 147	131	2.6 ± 1.3	164	38.3 °C ± 0.1^a^	129	6 (20%)
	2 section	988 ± 200^a^	64	9.3 ± 4.7	159			
	3 section	3176 ± 750^a^	75	19.6 ± 6.6^a^	106			
	4 section	5213 ± 484^a^	29	16.8 ± 4.6^a^	87			
	5 section	3910 ± 649^a^	52	7.1 ± 3.6	161			
	6 section	2942 ± 477^a^	51	4.0 ± 2.8	228			
	Total Hemisphere	2764 ± 256^a^	29	9.9 ± 2.9^a^	92

**Table 2:** Assessment of brain edema, NSS, subarachnoid hemorrhage and BBB breakdown in non-injured hemisphere. ^a^ = *p* < 0.01

Groups		BBB *n* = 10		Brain Edema *n* = 10		NSS *n* = 20		Subarachnoid hemorrhage n (%), *n* = 20
		Mean and SEM	Variability%	Mean and SEM %	Variability%	Mean and SEM	Variability%	
Naive	1 section	63 ± 24	120	−1.4 ± 0.9	675	1 ± 0.3	97	(0%)
	2 section	120 ± 17	44	1.3 ± 2.9	714			
	3 section	203 ± 26	41	1.9 ± 2.3	382			
	4 section	127 ± 27	67	1 ± 4	1324			
	5 section	191 ± 31	52	1.6 ± 2.9	584			
	6 section	139 ± 25	56	−0.4 ± 2.1	− 1883			
	Total Hemisphere	141 ± 14	31	0.7 ± 1.2	573			
Laser 25jx1	1 section	44 ± 36	257	0.9 ± 2.3	780	2 ± 0.5	89	(0%)
	2 section	109 ± 24	69	0.7 ± 2.1	914			
	3 section	197 ± 38	61	−1.9 ± 2.9	− 422			
	4 section	241 ± 63	82	1.7 ± 2.1	408			
	5 section	224 ± 47	66	2.2 ± 2.2	324			
	6 section	171 ± 27	50	7 ± 1.7	77			
	Total Hemisphere	172 ± 16	29	1.8 ± 0.6	115			
Laser 50jx1	1 section	79 ± 51	203	2.8 ± 2.1	234	3 ± 0.6	108	(0%)
	2 section	145 ± 53	117	0.7 ± 3.2	1529			
	3 section	150 ± 29	61	2 ± 2.6	415			
	4 section	233 ± 83	112	1.6 ± 3.5	696			
	5 section	221 ± 96	136	2.4 ± 2.7	359			
	6 section	257 ± 124	153	2.8 ± 2.9	327			
	Total Hemisphere	173 ± 55	101	2 ± 1.1	175			
Laser 50jx2	1 section	132 ± 42	101	2.4 ± 1.4	182	5 ± 0.9^a^	81	(0%)
	2 section	186 ± 78	132	1.7 ± 2.4	431			
	3 section	146 ± 2	56	1.3 ± 3	719			
	4 section	230 ± 51	70	0.7 ± 2.7	1170			
	5 section	304 ± 94	98	1.3 ± 2.4	598			
	6 section	166 ± 23	44	1.1 ± 2.6	758			
	Total Hemisphere	188 ± 40	68	1.4 ± 1.4	319			
Laser 50jx5	1 section	271 ± 73^a^	86	5.3 ± 1.7	101	13 ± 1.1^a^	39	(0%)
	2 section	437 ± 172^a^	124	6.3 ± 2.5	128			
	3 section	497 ± 210	134	4.5 ± 3	208			
	4 section	591 ± 285^a^	152	−0.8 ± 3.6	− 1350			
	5 section	432 ± 102	75	3.3 ± 2.7	258			
	6 section	293 ± 73	78	4.7 ± 2.2	157			
	Total Hemisphere	420 ± 94^a^	71	3.8 ± 1.1	92			
Laser 50jx10	1 section	456 ± 89^a^	62	7 ± 1.7	76	16 ± 1.1^a^	30	(0%)
	2 section	572 ± 129^a^	71	3.6 ± 1.3	111			
	3 section	759 ± 273^a^	114	4.3 ± 1.4	100			
	4 section	658 ± 117^a^	56	2.2 ± 1.3	180			
	5 section	485 ± 42^a^	27	2.4 ± 2.9	381			
	6 section	448 ± 70^a^	50	0.8 ± 0.7	264			
	Total Hemisphere	563 ± 66^a^	37	3.4 ± 0.6	58			
t-MCAO	1 section	147 ± 92	198	12.3 ± 5.3^a^	135	19 ± 1.3^a^	34	5^a^ (20%)
	2 section	410 ± 101^a^	78	6.1 ± 3.1	158			
	3 section	1001 ± 350^a^	111	4.6 ± 2.4	165			
	4 section	1643 ± 387^a^	74	6 ± 2.4	124			
	5 section	1179 ± 163	44	2.1 ± 2.1	314			
	6 section	1331 ± 179	42	2.4 ± 2.5	334			
	Total Hemisphere	952 ± 184^a^	61	5.6 ± 2.2	122			
p-MCAO	1 section	196 ± 118	191	13.2 ± 4.6^a^	109	20 ± 1.5^a^	37	5^a^ (20%)
	2 section	553 ± 171^a^	98	12.9 ± 3.7^a^	90			
	3 section	944 ± 159^a^	53	7.1 ± 3.9^a^	173			
	4 section	7991 ± 334^a^	53	7.4 ± 4.6	199			
	5 section	1813 ± 304^a^	53	1.6 ± 2.7	537			
	6 section	1559 ± 256^a^	52	0.1 ± 2.5	12,173			
	Total Hemisphere	1176 ± 224^a^	45	7 ± 2.6^a^	115			

### Mortality

The mortality rate was 17 and 20% at MCAO-p and MCAO-t subgroups respectively, while there was 0% mortality in all laser subgroups. The SAH rate was 20% at each of the MCAO subgroups and 0% in all laser subgroups (see Table 1).

### Mean Body Temperature

Mean body temperature was elevated to levels of 38.2 C and 38.3 C in transient and permanent MCAO subgroups respectively, while there was an only slight elevation of mean body temperature to levels of 37.3 C and 37.4 C in laser subgroups, almost similar to mean temperature of 37.2 C in naïve control subgroup. The variability of body temperatures was significantly elevated to 121 and 129% at MCAO subgroups in comparison to all laser subgroups and naïve control subgroup. Apart from 50 joules X 10 laser subgroup, which has higher variability of 84% (but which was also lower than in MCAO subgroups), all laser subgroups had the same or slightly lower variability of temperature in comparison to 59% in rats at naïve control subgroup (see Table 1).

### BBB Breakdown

During the trial, a significant increase in BBB breakage was observed at the injured hemisphere in all laser subgroups of 50 joules in comparison to naïve rats at control subgroup. Conversely, in the non–injured hemisphere only laser subgroups of 50 joules X 5 and 50 joules X 10 caused a significant BBB breakage in comparison to the control subgroup. Laser subgroups with lower irradiation power did not show a significant increase in BBB breakage at the non–injured hemisphere in comparison to the control subgroup. These findings match the observation we made in the study design that laser power less than 50 joules X 5 was not suitable for stroke induction at a level comparable to induction by MCAO in a rat model, although it can also cause brain injury in milder strokes.

BBB breakage at non-injured hemisphere was significantly lower at laser subgroups of 50 joules X 5 and 50 joules X 10 in comparison to transient and permanent MCAO subgroups. BBB breakage at injured hemisphere was lower at laser subgroups of 50 joules X 5 and 50 joules X 10 in comparison to permanent MCAO subgroup, but only laser subgroup of 50 X 5 joules showed lower BBB breakage in comparison to MCAO transient subgroup. The BBB breakage was higher at Laser 50 X 10 subgroup than in transient MCAO subgroup. There were no significant differences in variability of BBB breakage between the subgroups (see Table 2 and Fig. 2 and Fig. 3).

### Infarct Zone

The infarct zone, measured 24 h after stroke induction, was significantly lower in laser subgroups of 50 joules X 5 and 50 joules X 10 in comparison to MCAO-p subgroup but not in comparison to MCAO-t subgroup. The variability of the infarct zone was significantly lower in all laser subgroups in comparison to both MCAO subgroups (see Fig. 4).

### The NSS Score

The mean NSS score in laser subgroups of 50 joules X 5 and 50 joules X 10 was 13 +/− 1.1 and 16 +/− 1.1 respectively. The mean NSS score in MCAO-t and MCAO-p subgroups was 19 +/− 1.3 and 20 +/− 1.5 respectively. There was no significant variability difference between the laser and the MCAO subgroups (see Table 2).

### Brain Edema

The extent of brain edema, measured 24 h after the stroke induction, was lower in laser subgroups of 50 joules X 5 and 50 joules X 10 in comparison to MCAO-t subgroup and significantly lower in laser subgroups of 50 joules X 5 and 50 joules X 10 in comparison to MCAO-p subgroup (see Fig. 5). The variability of brain edema was significantly lower in all laser subgroups in comparison to both transient and permanent MCAO subgroups (see Fig. 4).

## Discussion

### Summary of Main Findings

Compared to original MCAO permanent or transient techniques, the model of laser-induced stroke demonstrated neither mortality nor SAH, findings that are favorable with the minimally invasive method we used. We can expect that null level of SAH and low mortality rate will be the rule for the laser model. The reason for this assumption is that the main cause of SAH and death in the MCAO induced stroke is tearing of a blood vessel and subsequent intracranial pressure (ICP) elevation. The laser impact on the brain tissue does not include direct blood vessel intervention and also causes coagulation, leading to a minimal risk of blood vessel tearing in the laser model. Additionally, as our findings suggest, laser-induced stroke causes less brain edema and less ICP elevation than the MCAO induced stroke, which also positively impacts the survival rates.

Temperature elevation was almost absent in rats with laser-induced stroke in comparison to rats with MCAO induced stroke, and the temperature variability at the laser subgroups was lower. The reason for these findings is that the laser method is relatively minimally invasive and does not cause hypothalamic artery occlusion, which often happens during the MCAO procedure and causes impairment of the hypothalamus function which includes the temperature control [8].

The low variability of the laser method was a consistent finding of our study and was also supported by the results of stroke size and extent of brain edema. Compared to the MCAO-p subgroup there was both less histological damage and less variability than in laser subgroups. The important finding of our study is that the impact of a laser can be less, the same, or even more than that of the artery occlusion, depending on the induced power, but the dispersion of its impact between different rats stays consistently lower. Therefore, it is suggested by these results that the laser method of inducible stroke in rats is precise and more controllable and has lower deviations at major outcomes of interest than the commonly-used MCAO method.

Another important finding was that the histological examination of brain tissue showed that in laser groups, the damage to the brain tissue occurred only in the region of motor cortex, without the involvement of the striatum area. These findings demonstrate an important advantage of the laser model which is completely absent in the MCAO model: the ability to induce localized strokes and small strokes. The laser technique permits the choice of a precise location and desired level of impact by pointing the beam and regulating its power. The MCAO technique lacks this ability, which limits the vessel size which can be occluded, and the actual area of its final impact depends on collateral vessels. The laser method can be effective in the induction of small and peripheral strokes, while MCAO method cannot.

The ability of laser beams to amplify at the chosen point allows also a unique opportunity to induce deep and defined strokes in the chosen areas of the brain. This model is more complicated and more expensive than the one we performed in our study but is possible, in contrast to the MCAO method which cannot induce isolated deep strokes. Future research will explore this assumption.

An important advantage of the laser method is its simplicity. Proper MCAO operation performance requires prolonged training, while a laser is easy to use. The costs of research using the laser method will be lower because highly trained staff is not required.

In the discussion of limitations of our study, we should mention the possibility that laser-induced stroke does not completely resemble an acute vascular occlusive stroke. The difference is that the laser irradiation of the brain generates immediate tissue scar in the site of its impact. This effect may not play a role in the peripheral area of the laser impact when damaged blood vessels fail to provide blood from the scar area to the surrounding tissue. Contrary to the central area, the peripheral area of the laser-induced stroke resembles the necrotic area of the vascular occlusive stroke. In summary, we can assume that laser-induced stroke is initially more organized, at least at its center, and actually resembles a vascular occlusive stroke that occurred several days or more ago. For this reason, laser stroke model can have limited value in testing drugs that aim to prevent stroke propagation but can be ideal for the research of isolated motor cortex stroke impact on prolonged motor, behavior and cognitive impairment.

### Limitations

An additional limitation is the relatively small number of rats whose brains were investigated for stroke size, brain edema extent, BBB breakage and presence of SAH. In our study, only half of the included rats from each subgroup underwent these full examinations, which limits the research power. Contrarily it should be emphasized that all rats underwent NSS evaluation and were followed for survival rate. All laser subgroups demonstrated less neurological impairment and lower mortality rate compared to MCAO subgroups, so there is a high probability that uninvestigated rats had the same reduction of stroke size, brain edema, BBB breakage and SAH, which enlarges our study power. This assumption can be proved in additional studies by a larger number of investigated brains or by performing MRI to all rats.

There are two reasons why we did not compare our model with other laser models of stroke described in the literature: 1. The degree of damage caused by the induction of stroke via existing laser models (such as by Rose Bengal) is relatively low. This makes it difficult to correctly assess the damage, including brain swelling, that may occur. 2. In the laser method for induction of ischemia, a craniotomy procedure is performed. The craniotomy procedure is very invasive and can lead to additional brain injury by increasing the permeability of BBB. This brain damage is not associated with stroke, and therefore it is impossible to adequately assess and compare the extent of brain damage that occurs in the groups.

Initially, we focused on creating a simple and reliable model suitable for behavioral assessment following traumatic brain damage or stroke, which is why we focused the laser directly to the motor cortex. Intraluminal occlusion of the middle cerebral artery is one of the most popular, reliable, and well-established animal models in the literature. The extent of damage in intraluminal middle cerebral artery occlusion remains the current gold standard for stroke models. The main outcomes of the laser model, including the extension of the cerebral edema, the volume of the infarction zone, the BBB breakdown, and the neurological deficit, approximately correspond to the degree of brain damage in the focal intraluminal middle cerebral artery occlusion technique. Therefore, the focal intraluminal middle cerebral artery occlusion model acts as an appropriate comparison for the laser model. The laser model has a practical application for neuro-behavioral assessment following brain injury. However, an extensive evaluation of the behavioral profile of the laser model was beyond the scope of the current study.

## Conclusion

In conclusion, the proposed model has advantages and disadvantages. Since the majority of human ischemic strokes are caused by thromboembolism, the drawback of the laser model is its impossibility to mimic human stroke more closely than other models. On the other hand, the laser model has a significant advantage on the commonly used MCAO model due to its extremely low variability of major outcomes of interest described above: low mortality rates, a unique ability to control the induced stroke size and location, its affordability, and its ease of use.

## Materials and Methods

### Aims

The purpose of our study was to establish and examine a novel model of laser-induced stroke with an extensive and isolated lesion of the cerebral cortex and compare the new model with the currently accepted model MCAO. In this article, we compared the outcomes of stroke size, brain edema volume, BBB breakdown extent, the severity of neurological impairment estimated by NSS score and rate of subarachnoid hemorrhage and mortality in rats subjected to stroke by two techniques: the common used MCAO method and novel laser method.

The experiments were conducted in accordance with the recommendation of the Declarations of Helsinki and Tokyo and the guidelines for the use of experimental animals of the European Community. The experiments were approved by the Animal Care Committee of Ben-Gurion University of Negev, Israel.

### Animals

A total of 210 male Sprague-Dawley rats (Harlan Laboratories, Israel) were bought for this experiment. Rats had no overt pathology and weighed 300 to 350 g each. Rats were kept in cages, with 3 rats per cage for at least 3 days to allow adaptation. Purina Chow and water were available ad libitum.

### Experimental Groups

Initially, rats were randomly assigned to laser groups (120 rats) and to control MCAO groups (90 rats). In the MCAO group, 30 rats were randomly assigned to each of the MCAO-t and MCAO-p research subgroups. An additional 30 rats were assigned to the control (sham) subgroup that underwent anesthesia and skin incision only, without MCAO catheterization. Because of the 17 and 20% percent of mortality rate at MCAO subgroups, the final number of rats was 25 at MCAO-t and 24 at MCAO-p subgroups respectively. In the laser group, 20 rats were randomly assigned to each of the five research subgroups that underwent laser irradiation with a different power of 25X1, 50X1, 50X2, 50X5 and 50X10 joules respectively. An additional 20 rats were assigned to the control (sham) subgroup that underwent anesthesia and skin incision only, without laser irradiation. The mortality rate in laser groups was 0% so the final number of rats remained unchanged.

### Experimental Design

The MCAO procedure was performed according to the method of Zea Longa [9]. In this research we used its modified version adapted for internal carotid artery (ICA) approach. This approach was chosen because it has been shown to produce lower variability in the infarct volume, better weight gain after surgery and reduced mortality [10]. The operation was performed under aseptic conditions in accordance with accepted principles in animal surgeries. Rats were anesthetized with a mixture of 2% isoflurane in oxygen and were allowed to breathe spontaneously. Core body temperature was maintained at 37 °C throughout the procedure with a rectal temperature-regulated heating pad. We used an internal carotid artery access model of MCAO. In this procedure, the right common carotid artery (CCA) was exposed through a midline neck incision and was carefully dissected from surrounding tissues, from its bifurcation to the base of the skull. The occipital artery and the branches of the ECA were then isolated and their branches were dissected and coagulated. The ECA was further dissected distally and coagulated along with the terminal lingual and maxillary artery branches. In the next step, the catheter was inserted via the ECA stump and directly through the ICA to achieve MCAO. The thread was then fixed by tying a silk filament over the ECA. In our technique, in contrast to alternative methods, the catheter was inserted directly through the ICA. Additionally, a 4–0 silk suture was tied loosely around the ICA immediately above the CCA bifurcation and proximal to filament insertion point. The purpose of this proximal ligation was to occlude the ICA while the additional distal ligation was used to reduce the bleeding around the filament and to secure it in a place. The suture has been left in place permanently and the incision was closed using surgical sutures. After this procedure, the anesthesia was discontinued, and rats returned to their cages for recovery. The duration of the entire surgery was approximately 25–30 min.

In the design of the laser protocol, we searched for an optimal level of laser irradiation which would not cause bone destruction. From the collected data, which is not presented in this article, we concluded that laser irradiation of 50 joules for one-second duration or less does not cause destruction at the bones of the skull. Another aspect of optimal laser protocol design was to determine the minimal laser irradiation power which causes brain infarction, BBB breakage and brain edema at a level comparable to the original MCAO protocol. From the collected data, which is not presented at this article, we concluded that laser irradiation power of 50X5 (joules X number of targets) or more has this comparable level of impact on the brain tissue, although even less power caused significant brain tissue impairment compared to the control group.

For the laser procedure rats were anesthetized with a mixture of 2% isoflurane in oxygen without tracheostomy and were allowed to breathe spontaneously. Core body temperature was maintained at 37 °C throughout the procedure with a rectal temperature-regulated heating pad. There were no differences in the time assigned for anesthesia between the groups. The rats were placed on a stereotaxic head holder in the prone position. A skin incision of three centimeters was made, the scalp was reflected laterally and the area between Bregma and Lambda was exposed and marked. Sharplan 3000 Neodymium-Y AG (Nd-YAG) laser has been approximated to induce its beam at a distance of two mm from the exposed area. Rats from the five research subgroups received laser irradiation with a different power of 25 or 50 joules and a different number of pulses: 1, 2, 5 or 10, one-second duration each, pointed to one of the marked targets above the right hemisphere between Bregma and Lambda areas (Fig. 1). Eventually, the rats from the five research subgroups were exposed to laser irradiation of 25X1, 50X1, 50X2, 50X5 and 50X10 (joules X number of targets) respectively. Afterwards the rats were removed from the device and the scalp was closed using surgical sutures. After this procedure, the anesthesia was discontinued, and rats were returned to their cages for recovery. The duration of the entire procedure was approximately 5 min.

All the rats were euthanized 24 h after the experiment. Brains of ten rats from each subgroup (Naïve, MCAO-t, MCAO-p, Laser 25X1, Laser 50X1, Laser 50X2, Laser 50X5, and Laser 50X10), which were chosen randomly, were further examined for determining the size of the infarcted brain, brain edema and BBB breakage extent.

### NSS Evaluation

At first, the animals were tested for the existence of the neurological deficits following the induced stroke and their motor deficits were graded on a cumulative scale from 0 to 4. This scoring method was used to identify and discard rats that did not develop motor deficit at one hour after laser or MCAO procedures. The score was calculated as follows: 0 - no visible neurological deficits; 1 - forelimb flexion, 2 - contralateral forelimb grips weakly (the operator places the animal on an absorbent pad and gently pulls the tail), 3 - circling to the paretic side only when pulled by the tail (the animal is allowed to move about freely on the absorbent pad) and 4 – spontaneous circling. The rats that developed any neurological impairment were tested further with more sophisticated NSS method consisted of 43 parameters with a possible score of 0 to 43 [11].

### Measurement of Brain Infarct Volume

In order to measure the extent of brain edema, the TTC staining method was performed 24 h after the operation. 10 rats from each experiment subgroup were euthanized by inspiration of high CO2 and were decapitated. Their brains were quickly isolated and sectioned into 6 coronal slices, each 2 mm of thickness. The set of slices from each brain were incubated for 30 min at 37 °C in 0.05% TTC. Following staining, the slices were scanned with an optical scanner (Canon Cano Scan 4200F; resolution 1600 × 1600dpi). The unstained areas of the fixed brain slices were defined as infarcted. The size of brain injury was measured by Image J 1.37v software, calculated in arbitrary units (pixels) and expressed as a percentage of the normal areas in the contralateral unaffected hemisphere. The total size of infarction was obtained by numeric integration of the area of marked pallor, measured in six consecutive 2 mm coronal sections. In order to correct for the tissue swelling factor, the following formula was utilized: corrected infarct size = infarct size × contralateral hemisphere size / ipsilateral hemisphere size. Infarcted volume had been expressed as a percentage of the total brain [12].

### Measurement of Brain Edema Extent

To assess the extent of right cerebral hemisphere edema, the volumes of both hemispheres had been calculated in the arbitrary units (pixels) from the summation of coronal slice areas using the Image J 1.37 software, after they were scanned with an optical scanner (Canon Cano Scan 4200F; resolution 1600X1600 dpi). Brain edema was expressed as a percentage of the normal areas in the contralateral unaffected hemisphere. The extent of swelling was calculated using Kaplan method: extent of edema = (the volume of right hemisphere – the volume of left hemisphere) / the volume of left hemisphere [13].

### Measurement of BBB Breakage Extent

In the current study, the timing for determination of BBB disruption was performed 24 h after laser and MCAO procedures. 10 rats from each experiment subgroup received Evans Blue 2% in saline (4 ml/kg), which was administered intravenously through the cannulated tail vein as a blood/brain permeability tracer and was allowed to circulate for 60 min. To remove the intravascularly localized dye, the rats’ chests were opened, and the animals were perfused with cooled saline through the left ventricle at a pressure of 110 mmHg until colorless perfusion fluid was obtained from the right atrium. Their brains were quickly isolated and sliced rostrocaudally into serial 2-mm-thick slices. Then the brain slices were divided into the left and right hemisphere and measurements of vascular permeability were made by comparing its weight with pre-weighed loci in the 6 slices.

Each brain area was weighted and homogenized in 1 ml of 50% trichloroacetic acid (weight/volume) and was centrifuged at 10,000×g for 20 min. One ml of the supernatant was added to 1.5 ml of the solvent (50% trichloroacetic acid/96% ethanol, 1:3). A fluorescence detector (model Infinite 200 PRO multimode reader; Tecan, Männedorf Switzerland) was used at an excitation wavelength of 620 nm (bandwidth 10 nm) and an emission wavelength of 680 nm (bandwidth 10 nm). Calculations were based on external standards in the solvent (10 ± 500 ng/ml). Data were expressed as mean ± SD (in mg/g of protein) of extravagated Evans Blue dye per gram of brain tissue [14].
